# Edible Coatings Fortified With Carvacrol Reduce *Campylobacter jejuni* on Chicken Wingettes and Modulate Expression of Select Virulence Genes

**DOI:** 10.3389/fmicb.2019.00583

**Published:** 2019-03-21

**Authors:** Sandip Shrestha, Basanta R. Wagle, Abhinav Upadhyay, Komala Arsi, Indu Upadhyaya, Dan J. Donoghue, Annie M. Donoghue

**Affiliations:** ^1^Department of Poultry Science, University of Arkansas, Fayetteville, AR, United States; ^2^Department of Animal Science, University of Connecticut, Storrs, CT, United States; ^3^School of Agriculture, Tennessee Tech University, Cookeville, TN, United States; ^4^Poultry Production and Product Safety Research Unit, United States Department of Agriculture-Agriculture Research Service, Fayetteville, AR, United States

**Keywords:** *Campylobacter jejuni*, carvacrol, gum arabic, chitosan, postharvest poultry, antibiotic alternative, gene expression, color

## Abstract

*Campylobacter jejuni*, a leading cause of foodborne disease in humans, associate primarily with consumption of contaminated poultry and poultry products. Intervention strategies aimed at reducing *C. jejuni* contamination on poultry products could significantly reduce *C. jejuni* infection in humans. This study evaluated the efficacy of gum arabic (GA) and chitosan (CH) fortified with carvacrol (CR) as an antimicrobial coating treatment for reducing *C. jejuni* on chicken wingettes. Aforementioned compounds are generally recognized as safe status compounds obtained from gum arabic tree, crustaceans and oregano oil respectively. A total of four separate trials were conducted in which wingettes were randomly assigned to baseline, saline control (wingettes washed with saline), GA (10%), CH (2%), CR (0.25, 0.5, or 1%) or their combinations. Each wingette was inoculated with a cocktail of four wild-type strains of *C. jejuni* (∼7.5 log_10_ cfu/sample). Following 1 min of coating in aforementioned treatments, wingettes were air dried (1 h) and sampled at 0, 1, 3, 5, and 7 days of refrigerated storage for *C. jejuni* and total aerobic counts (*n* = 5 wingettes/treatment/day). In addition, the effect of treatments on wingette color was measured using a Minolta colorimeter. Furthermore, the effect of treatments on the expression of *C. jejuni* survival/virulence genes was evaluated using real-time quantitative PCR. Results showed that all three doses of CR, CH or GA-based coating fortified with CR reduced *C. jejuni* from day 0 through 7 by up to 3.0 log_10_ cfu/sample (*P* < 0.05). The antimicrobial efficacy of GA was improved by CR and the coatings reduced *C. jejuni* by ∼1 to 2 log_10_ cfu/sample at day 7. Moreover, CH + CR coatings reduced total aerobic counts when compared with non-coated samples for a majority of the storage times. No significant difference in the color of chicken wingettes was observed between treatments. Exposure of pathogen to sublethal concentrations of CR, CH or combination significantly modulated select genes encoding for energy taxis (*cetB*), motility (*motA*), binding (*cadF*), and attachment (*jlpA*). The results suggest that GA or CH-based coating with CR could potentially be used as a natural antimicrobial to control *C. jejuni* in postharvest poultry products.

## Introduction

*Campylobacter jejuni* infection in humans continues to be a significant public health problem throughout the world (Crim et al., 2015; Mangen et al., 2016). In the United States, *Campylobacter* causes 1.3 million illnesses each year and contaminated chicken meat is considered as one of the primary sources of *Campylobacter* infection in humans (Crim et al., 2015). Epidemiological studies have shown that up to 70–80% of retail raw chicken meat in the United States is contaminated with *Campylobacter* (Cui et al., 2005). Recently, the incidence of *Campylobacter* in the United States surpassed that of *Salmonella* (17.43 vs. 16.66 per 100,000 people; Marder et al., 2017). The high level, 10^7^ cfu/g of cecal content, of *Campylobacter* in the ceca of market age birds (Rudi et al., 2004) leads to potential carcass contamination at processing plants thereby posing a serious public health threat. The concerns are further raised due to the low infective dose (∼500 cells; Kothary and Babu, 2001) required to cause infection in humans and potentially fatal sequelae such as Guillain-Barré syndrome (Hughes and Cornblath, 2005).

Conventional poultry processing constitutes several steps (scalding, picking, evisceration, chilling) that reduces but does not eliminate *Campylobacter* contamination on carcasses (Elvers et al., 2011; Alonso-Hernando et al., 2013). In addition, poultry processors in the United States heavily rely on the use of inorganic/synthetic chemicals, such as peracetic acid, trisodium phosphate and chlorine-based compounds to reduce poultry carcass contamination (Keener et al., 2004; Oyarzabal, 2005; McKee, 2011). However, with increasing consumer demand for safe, natural and minimally processed foods, the use of natural, plant-derived antimicrobials with generally recognized as safe (GRAS) status is gaining attention for improving safety of poultry products. These compounds are naturally derived metabolites and/or by-products from various plants sources and have been used as the chief source of antimicrobials in human medicine for thousands of years (Fabricant and Farnsworth, 2001). The antimicrobial activity of several plant-derived compounds has been documented (Cowan, 1999; Savoia, 2012). Carvacrol (CR; 5-isopropyl-2-methylphenol) is a polyphenolic compound which is present in the essential oil fractions of oregano (60–74% carvacrol) and thyme (45% carvacrol) (Lagouri et al., 1993). Studies have shown that this compound has significant antibacterial properties against a wide range of foodborne pathogens including *Salmonella* spp. (Kim et al., 1995; Kollanoor Johny et al., 2010; Mattson et al., 2011), *Campylobacter jejuni* (Ravishankar et al., 2008), *Listeria monocytogenes* (Upadhyay et al., 2015), *Escherichia coli* O157:H7 (Du et al., 2008), and *Bacillus cereus* (Ultee et al., 2002). Carvacrol is currently listed as GRAS by the U.S. Food and Drug Administration (Code of Federal Regulations 21 part 172).

Over the last decade, significant research on the use of antimicrobial films or coating materials for improving microbiological safety and shelf-life of food products has been undertaken (Dutta et al., 2009; Valencia-Chamorro et al., 2011). The application of antimicrobial edible coatings onto the surface of raw poultry carcass could be an alternative to reduce foodborne pathogens including *Campylobacter* on poultry products. Antimicrobial edible coatings, due to their presence on products, reduces the chance of cross-contamination during storage and handling. Gum arabic (GA) is obtained from the gum arabic tree (*Acacia senegal* or *Senegalia senegal*) and is composed of a highly branched arrangement of simple sugars galactose, arabinose, rhamnose, and glucuronic acids (Phillips, 1998; Kennedy et al., 2012). The mixture of polysaccharides and glycoproteins gives GA the properties of a glue and binder which is edible for humans. Since it is safe for human consumption, it is one of the most commonly used natural coatings for various food surfaces and has been used in variety of food preparations such as soft drink syrup, hard gummy candies, marshmallows and nougats (Phillips, 1998; Dauqan and Abdullah, 2013; Patel and Goyal, 2015; Food and Drug Administration [FDA], 2018). Pharmaceutical drugs and cosmetics also use the gum as a binder, emulsifying agent and a thickening agent (Kennedy et al., 2012). In addition, wine makers have used GA as a wine fining agent (Kennedy et al., 2012). Recently, the use of GA as a coating to improve shelf-life as well as safety of different food products has been studied. Ali et al. (2010) found that 10% GA coating enhanced shelf-life and improved postharvest quality of tomatoes. Upadhyaya et al. (2016) found that 10% GA fortified with different phytochemicals (carvacrol, eugenol or *β*-resorcylic acid) significantly reduced *Salmonella* Enteritidis counts on shell eggs. Similarly, chitosan (CH), a polysaccharide obtained from crustaceans, is another compound that has been extensively studied as an antimicrobial coating on food products and employed as an effective antimicrobial for reducing various foodborne pathogens including *Listeria monocytogenes* (Upadhyay et al., 2015), *Salmonella* Typhimurium (Menconi et al., 2013) and *Campylobacter jejuni* (Woo-Ming, 2015). The CH-based coatings are non-toxic, non-polluting, biodegradable, edible, and are easy to use in industry setting (Aider, 2010; Kong et al., 2010; Sánchez-González et al., 2011). Both GA and CH are classified as GRAS by the U.S. FDA for use in foods (Code of Federal Regulations 21 part 184, 170).

The aim of the present study was to investigate the anti-*Campylobacter* effect of 10% GA or 2% medium molecular weight (MMW) CH coatings fortified with CR (0.25, 0.5, and 1%) on inoculated chicken wingettes. In addition, the effect of the aforementioned treatments on the color of chicken wingettes was evaluated. The effect of select treatments on the expression of genes critical for the survival of *C. jejuni* in the environment and virulence was also investigated.

## Materials and Methods

### *C. jejuni* Inoculum Preparation

Four wild-type strains (S-1, S-3, S-4, and S-8) of *C. jejuni*, previously isolated from commercial broilers by our laboratory were used in this experiment. *C. jejuni* inoculum was prepared as described by Shrestha et al. (2017). Briefly, one loopful of glycerol stock of the wild-type strain *C. jejuni* was inoculated into 5 mL of *Campylobacter* Enrichment Broth (CEB; Catalog No. 7526A, Neogen Corp, Lansing, MI, United States) and incubated at 42°C in a microaerophilic atmosphere (5% O_2_, 10% CO_2_, and 85% N_2_) for 48 h. Each strain was sub-cultured again at the same temperature and atmospheric conditions for 24 h. Sub-cultured *C. jejuni* was centrifuged at 3500 × *g* for 10 min, the supernatant discarded and the cell pellet from each strain was mixed and resuspended in required volume of Butterfield’s Phosphate Diluent (BPD; 0.625 mM potassium dihydrogen phosphate, pH 6.67). The resulting suspension was used as inoculum (final bacterial concentration was ∼8.5 to 9.0 log_10_ cfu/mL). The bacterial count in the four-strain cocktail was confirmed by plating 100 μL of culture suspension and its 10-fold dilution on *Campylobacter* line agar (CLA; Line, 2001) followed by incubation at 42°C in a microaerophilic atmosphere for 48 h.

### Wingette Sample Preparation

Chicken wings were procured from the University of Arkansas Poultry Processing Plant (Fayetteville, AR, United States). The middle portion (wingette) of the whole wing was separated by cutting on shoulder and elbow joints. Separated wingettes were stored at -20°C until the day of experiment.

### CR Suspension/Wash Treatment Preparation

Carvacrol (Catalog No. W224502, Sigma-Aldrich Co., St. Louis, MO, United States) was suspended in appropriate volume of sterile BPD solution to obtain 0.25, 0.5, 1% CR suspension in BPD. The suspension was stirred at 300 rpm for 15 min before loading to sterile Whirl-Pak^TM^ bag (Catalog No. 018126C, Nasco, Fisher Scientific, Suwanee, GA, United States).

### Coating Treatment Preparation

The GA (Catalog No. G9752, Sigma-Aldrich CO., St. Louis, MO, United States) coating solution was prepared based on previously published article (Upadhyaya et al., 2016) with slight modification. In brief, 10 g of GA were added to 100 mL of sterile BPD. The solution was then stirred for 1 h at room temperature. Similarly, 2% MMW CH (190 – 310 kDa) (Catalog No. 448877, Sigma-Aldrich, St. Louis, MO, United States) solution was prepared by using the method developed by Woo-Ming (2015). Briefly, 2 g of MMW CH powder was dissolved in 100 mL of 50 mM acetic acid (Catalog No. A38C212, Fisher Scientific, Fair Lawn, NJ, United States) solution by stirring overnight at room temperature. To these coating solutions, required amounts of 100% CR were added and mixed continuously for 12 h at room temperature to prepare 0.25, 0.5, and 1% CR coating treatments.

### Evaluation of Antimicrobial Activity of Coating Treatments on Chicken Wingettes

Coatings of the chicken wingettes was based on the protocol from our laboratory (Woo-Ming, 2015; Wagle et al., 2018). For each coating materials, two independent trials were conducted using chicken wingettes from different batches. In each trial with GA, 225 thawed wingettes were individually inoculated with 50 μL (∼7.5 log_10_ cfu/sample) *C. jejuni* and were allowed to adhere for 30 min. Wingettes were randomly divided into nine different treatment groups which included baseline, BPD (wash control), 10% GA (coating control), CR (0.25, 0.5 and 1%) or CR (0.25, 0.5 and 1%) + 10% GA. Each wingette was individually placed in a sample bag containing 10 mL of the respective treatment for coating/dipping. Working with one treatment group at a time, wingettes were vigorously shaken/massaged for 1 min to obtain a complete coating. After coating/dipping, wingettes were removed from bags and allowed to dry for 1 h (30 min on each side). Wingettes were divided into sampling times (*n* = 5/treatment/time) at days 0, 1, 3, 5, or 7. To process the day 0 samples, microbial analysis was done immediately after drying while other wingettes were vacuum sealed by a commercially available vacuum sealer (Ziploc^®^ V201, Lake Barrington, IL, United States) and store at 4°C until the day of sampling. The antimicrobial effect of CH coating with CR was evaluated as described above. In each trial, the treatments were baseline, BPD (wash/dip control), 50 mM acetic acid (CH control), 2% CH (coating control), CR (0.25%, 0.5% and 1%) or CR (0.25%, 0.5% and 1%) + 2% CH. For each treatment, 5 wingettes were tested per time point (*n* = 5/treatment/time).

### Microbial Analysis

For all experiments, the samples were individually removed from vacuum packaging and aseptically transferred to a stomacher^®^ 400 classic bag (Catalog No. BA6041, Steward Ltd., Worthing, West Sussex, United Kingdom) containing 30 mL of Dey-Engley neutralizing broth (Catalog No. C7371, Hardy diagnostic, Santa Maria, CA, United States) followed by blending for 30 s at 250 rpm (Stomacher^®^ 400 Circulator, Steward Ltd., Worthing, West Sussex, United Kingdom). For all samples 10-fold dilutions were prepared from initial dilution in sterile BPD. Each dilution was surface plated onto CLA followed by incubation at 42°C microaerophilic condition for 48 h. For aerobic bacterial enumeration each dilution was plated onto tryptic soy agar (TSA; Catalog No. DF0369176, Becton, Dickinson and Company, Sparks, MD, United States) followed by incubation at 37°C under aerobic condition for 24 h. Bacterial colonies were counted and expressed as cfu/sample.

### Color Analysis

For the color analysis, a separate batch of chicken wingettes not inoculated with *C. jejuni* was allocated and subjected to similar treatments as described above. Color of wingettes was measured as described by Wagle et al. (2017a). International Commission on Illumination (CIE) L^∗^ (lightness), a^∗^ (redness), and b^∗^ (yellowness) values for each wingette were evaluated using a Minolta colorimeter (CR-300, Konica Minolta Sensing Inc., Japan). The instrument was calibrated against a white tile before the measurements. Three readings were taken on the lateral surface of each wingette, averaged and analyzed.

### RNA Extraction, cDNA Synthesis, and Real-Time Quantitative PCR

The effect of sub-inhibitory concentrations of CR (0.002%), CH (0.0125%) or CR (0.002%) + CH (0.0125%) combination on expression of selected virulence genes of *C. jejuni* was determined using a previously published method (Wagle et al., 2017a) with slight modification. Briefly, frozen whole chicken carcasses were obtained from the University of Arkansas poultry pilot processing plant (Fayetteville, AR, United States) and thawed at 4°C for 12 h. The meat exudate was collected into sterile centrifuged tubes followed by centrifugation at 3,000 × *g* for 20 min. The debris were removed, and the juice was filter sterilized with different size filters (0.8, 0.45 and finally through 0.2-μm cellulose acetate membrane [Catalog No. 28151261 (0.8-μm), 10035088 (0.4-μm), 14224474 (0.2-μm), VWR International, West Chester, PA, United States). *C. jejuni* S-8 was incubated in chicken juice with or without sub-inhibitory concentrations of CR, CH, or CH + CR at room temperature for approximately 1 h under aerobic condition. Total RNA was extracted using RNA mini kit (Catalog No. 12183018A, Invitrogen, Carlsbad, CA, United States). DNase treatment (Catalog No. 18068015, Thermo Fisher Scientific, Carlsbad, CA, United States) was done followed by the complementary DNA (cDNA) preparation using iScript cDNA synthesis kit (Catalog No. 1708890, Bio-Rad Laboratories, Inc., Hercules, CA, United States). All the primers in this study (Table 1) were designed from published Gene Bank *C. jejuni* sequences using Primer 3 software (National Center for Biotechnology Information) and obtained from Integrated DNA Technologies. The cDNA was used as the template for PCR reaction and the amplified product was detected by SYBR Green reagent (Catalog No. 1708880, iQ SYBR Green Supermix, Bio-Rad). Data were normalized to endogenous control (16S rRNA) and expression of candidate genes were analyzed using comparative critical threshold method on Quant Studio 3 real-time PCR system (Applied Biosystem, Thermo Fisher).

**Table 1:** Primers used for gene expression analysis using real-time quantitative PCR.

Gene with Accession no.	Primer	Sequence (5′-3′)	Gene description
16S-rRNA (NC_002163.1) (product length 78 bp)	Forward Reverse	5′-ATAAGCACCGGCTAACTCCG-3′ 5′-TTACGCCCAGTGATTCCGAG-3′	Ribosomal RNA (housekeeping gene)
*motA* (NC_002163.1) (product length 75 bp)	Forward Reverse	5′-AGCGGGTATTTCAGGTGCTT-3′ 5′-CCCCAAGGAGCAAAAAGTGC-3′	Flagellar motor protein
*motB* (NC_002163.1) (product length 51 bp)	Forward Reverse	5′-AATGCCCAGAATGTCCAGCA-3′ 5′-AGTCTGCATAAGGCACAGCC-3′	Flagellar motor protein
*fliA* (NC_002163.1) (product length 56 bp)	Forward Reverse	5′-AGCTTTCACGCCGTTACGAT-3′ 5′-TCTTGCAAAACCCCAGAAGT-3′	Flagella biosynthesis RNA polymerase sigma protein
*cetB* (NC_002163.1) (product length 88 bp)	Forward Reverse	5′-GCCTTGTTGCTGTTCTGCTC-3′ 5′-TTCCGTTCGTCGTATGCCAA-3′	Energy taxis protein/motility
*cadF* (NC_002163.1) (product length 135 bp)	Forward Reverse	5′-CGCGGGTGTAAAATTCCGTC-3′ 5′-TCCTTTTTGCCACCAAAACCA-3′	Outer-membrane fibronectin-binding protein
*ciaB* (NC_002163.1) (product length 50 bp)	Forward Reverse	5′-TCTCAGCTCAAGTCGTTCCA-3′ 5′-GCCCGCCTTAGAACTTACAA-3′	Invasion antigen protein
*jlpA* (NC_002163.1) (product length 66 bp)	Forward Reverse	5’-AGCACACAGGGAATCGACAG-3’ 5’-TAACGCTTCTGTGGCGTCTT-3	Surface exposed lipoprotein
*racS* (NC_002163.1) (product length 79 bp)	Forward Reverse	5′-AGACAAGTTGCCGAAGTTGC-3′ 5′-AGGCGATCTTGCCTACTTCA-3′	Two-component sensor/histidine kinase


### Statistical Analysis

The bacterial counts were log_10_ transformed (log_10_ cfu/sample) for analysis to achieve homogeneity of variance (Byrd et al., 2001). For the gene expression analysis, data were pooled and expressed as log_10_ of relative quantification (RQ) and were analyzed using ANOVA with the PROC MIXED procedure in SAS statistical software, version 9.3 (SAS Institute Inc., Cary, NC, United States). Means were partitioned by LSMEANS analysis, and a *P* < 0.05 was required for statistical significance.

## Results

### Efficacy of GA-Based or CH-Based Coating Treatments (With or Without CR) in Reducing *C. jejuni* on Chicken Wingettes

Table 2 shows the effect of 10% GA coating alone or fortified with CR (0.25, 0.5, or 1%) in reducing *C. jejuni* on chicken wingettes. The *C. jejuni* counts recovered from the baseline group (inoculated wingettes not subjected to any treatment) ranged from ∼6.3 to 7.0 log_10_ cfu/sample. In both trials, samples washed with the BPD control showed significant reduction (∼1 log_10_ cfu/sample) of *C. jejuni* counts from day 0 through day 7, when compared to the baseline group. As BPD has no anti-*Campylobacter* property, the observed reduction was probably due to washing away of loosely attached *C. jejuni* cells by the solution. Coating with the 10% GA showed consistent reduction (*P* < 0.05) in *C. jejuni* counts when compared to the baseline group, however, did not show significant difference with the BPD control in both the trials. All the tested doses of CR (0.25, 0.5, or 1%) significantly reduced *C. jejuni* counts from day 0 to day 7 in both the trials when compared with the BPD control. There was significant difference in anti-*Campylobacter* efficacy between 0.25% CR and 1% CR in trial 1, however, the results were not consistent between trials. The combination groups of 10% GA with different doses of CR (0.25, 0.5, or 1%) consistently reduced *C. jejuni* counts at all days in both trials when compared to the 10% GA control (*P* < 0.05). For example, addition of 1% CR in GA produced additional reduction in *C. jejuni* counts by ∼1.8 log_10_ cfu/sample (trial 1) and ∼1.2 log_10_ cfu/sample (trial 2) at day 0 as compared to the GA alone. The difference in antimicrobial efficacy persisted during the storage period between GA and combination treatments. The anti-*Campylobacter* efficacy of GA + CR coating treatments was similar on majority of storage time points in both trials when compared with the respective doses of CR alone.

**Table 2:** The efficacy of gum arabic (GA), carvacrol (CR) and their combinations on survival of *Campylobacter jejuni* on chicken wingettes^1^.

Trial	Treatments	Day 0	Day 1	Day 3	Day 5	Day 7
1	Baseline	7.00 ± 0.03^a^	6.66 ± 0.03^a^	6.51 ± 0.03^a^	6.60 ± 0.03^a^	6.30 ± 0.11^a^
	BPD control	6.11 ± 0.07^b^	5.53 ± 0.07^b^	5.56 ± 0.08^b^	5.53 ± 0.03^b^	5.55 ± 0.08^b^
	10% GA	5.92 ± 0.05^b^	5.77 ± 0.10^b^	5.78 ± 0.09^b^	5.71 ± 0.02^b^	5.41 ± 0.07^b^
	0.25% CR	4.80 ± 0.14^c^	4.44 ± 0.17^c^	4.46 ± 0.12^cd^	4.60 ± 0.16^c^	4.69 ± 0.11^c^
	0.5% CR	4.00 ± 0.18^de^	4.10 ± 0.15^cd^	3.96 ± 0.17^e^	4.03 ± 0.14^de^	4.20 ± 0.06^cd^
	1% CR	3.62 ± 0.31^e^	3.81 ± 0.31^d^	2.82 ± 0.07^f^	3.41 ± 0.43^f^	3.59 ± 0.10^e^
	0.25% CR + 10% GA	4.85 ± 0.10^c^	4.70 ± 0.19^c^	4.80 ± 0.07^c^	4.48 ± 0.11^cd^	4.72 ± 0.16^c^
	0.5% CR + 10% GA	4.24 ± 0.18^d^	4.30 ± 0.36^cd^	4.24 ± 0.21^de^	4.27 ± 0.19^cd^	4.57 ± 0.22^c^
	1% CR + 10% GA	4.14 ± 0.21^d^	3.80 ± 0.29^d^	4.25 ± 0.12^de^	3.48 ± 0.27^ef^	3.78 ± 0.42^de^
2	Baseline	7.00 ± 0.09^a^	6.74 ± 0.04^a^	6.93 ± 0.05^a^	6.80 ± 0.07^a^	6.84 ± 0.09^a^
	BPD control	6.26 ± 0.04^b^	5.89 ± 0.05^b^	5.80 ± 0.04^b^	5.95 ± 0.09^b^	5.86 ± 0.08^b^
	10% GA	6.45 ± 0.09^b^	5.93 ± 0.05^b^	6.16 ± 0.06^b^	6.08 ± 0.09^b^	6.06 ± 0.06^b^
	0.25% CR	5.39 ± 0.14^de^	4.63 ± 0.48^c^	5.14 ± 0.19^c^	5.00 ± 0.21^cd^	5.33 ± 0.06^cd^
	0.5% CR	5.61 ± 0.17^cd^	4.96 ± 0.22^c^	4.92 ± 0.21^cd^	5.08 ± 0.21^cd^	5.13 ± 0.12^cd^
	1% CR	5.19 ± 0.06^e^	4.60 ± 0.29^c^	4.68 ± 0.37^cd^	4.70 ± 0.21^d^	5.01 ± 0.16^d^
	0.25% CR + 10% GA	5.80 ± 0.14^c^	5.21 ± 0.05^c^	5.17 ± 0.17^c^	5.42 ± 0.08^c^	5.38 ± 0.14^c^
	0.5% CR + 10% GA	5.31 ± 0.09^de^	5.15 ± 0.26^c^	5.12 ± 0.19^c^	5.33 ± 0.18^c^	5.16 ± 0.16^cd^
	1% CR +10% GA	5.20 ± 0.17^e^	5.01 ± 0.20^c^	4.50 ± 0.27^d^	5.24 ± 0.15^c^	5.04 ± 0.19^cd^


*^*1*^n = 5 replicates per treatment per day per trial. Values (log_*10*_ cfu/sample) presented as mean ± standard error of the mean. Within the same trial, within column with no common superscript differ significantly (*P* < 0.05).*

In an attempt to test the effect of different edible coatings fortified with CR, further testing was done with 2% CH as a coating material (Table 3). As shown in Table 3, the number of *C. jejuni* recovered from the baseline group ranged from ∼6.1 to 6.7 log_10_ cfu/sample in trial 1 and ∼6.6 to 7.3 log_10_ cfu/sample in trial 2. The BPD control significantly (*P* < 0.05) reduced the *C. jejuni* counts by ∼1 log_10_ cfu/sample at days 3 and 5 in trial 1, whereas in trial 2, the counts were significantly reduced throughout the sampling days when compared to the baseline. The number of *C. jejuni* recovered from skin samples treated with the 50 mM acetic acid solution (with adjusted pH ∼6.4) was not significantly different when compared with the *C. jejuni* counts recovered from samples washed with the BPD (except at day 1 in trial 2). The 2% CH coating consistently reduce *C. jejuni* counts in both trials as compared to baseline counts. When compared with its control (50 mM acetic acid solution), the 2% CH consistently reduced *C. jejuni* counts by ∼1 to 1.5 log_10_ cfu/sample, except at day 0 in trial 2 (*P* < 0.05). As observed in Table 2, all the tested doses of CR (0.25, 0.5, and 1%) significantly reduced *C. jejuni* counts from day 0 through 7 in both trials when compared with the BPD control (*P* < 0.05). The combination treatments were more effective than CH alone at select time points, however, a consistent improvement in the antimicrobial efficacy was not observed. For example, all the combination treatments were more effective than CH at day 1 in trial 1 and at day 0 in trial 2. However, by day 7, the combination treatments and CH coating were similar in their efficacy in reducing *C. jejuni.* A similar pattern was observed when the combination treatments were compared with CR. Only select combination treatments showed increased efficacy as compared to CR alone at various stages during refrigerated storage. For example, the combination of lowest dose (0.25%) of CR and CH increased antimicrobial efficacy by ∼0.5 log as compared with the 0.25% CR at days 3 and 7 in trial 1 and days 1 and 7 in trial 2. The 0.5% CH + CR combination was more effective than 0.5% CR treatment at days 1, 5, and 7 in trial 1 and at days 1 and 5 in trial 2. The combination of highest dose (1%) of CR with CH had increased efficacy at days 1 and 3 in trial 1; however, had decreased efficacy at day 3 in trial 2. The combination treatment was similar to corresponding CR treatments on rest of the storage days in both trials.

**Table 3:** The efficacy of chitosan (CH), carvacrol (CR) and their combinations on survival of *Campylobacter jejuni* on chicken wingettes^1^.

Trial	Treatments	Day 0	Day 1	Day 3	Day 5	Day 7
1	Baseline	6.65 ± 0.19^a^	6.31 ± 0.21^a^	6.64 ± 0.07^a^	6.11 ± 0.15^a^	6.71 ± 0.13^a^
	BPD control	6.06 ± 0.14^a^	5.89 ± 0.14^a^	5.73 ± 0.21^b^	5.34 ± 0.07^b^	6.26 ± 0.02^ab^
	50 mM acetic acid	6.02 ± 0.15^a^	6.11 ± 0.10^a^	5.59 ± 0.18^bc^	5.47 ± 0.09^b^	6.03 ± 0.13^b^
	2% CH	4.99 ± 0.24^b^	5.15 ± 0.12^b^	4.69 ± 0.23^de^	4.49 ± 0.33^cd^	4.69 ± 0.19^de^
	0.25% CR	4.56 ± 0.09^bc^	4.83 ± 0.12^bc^	5.04 ± 0.10^cd^	4.51 ± 0.08^cd^	5.43 ± 0.13^c^
	0.5% CR	4.56 ± 0.18^bc^	5.19 ± 0.09^b^	4.62 ± 0.19^def^	4.68 ± 0.14^c^	4.95 ± 0.36^cd^
	1% CR	4.08 ± 0.23^c^	5.16 ± 0.11^b^	5.12 ± 0.16^cd^	4.07 ± 0.21^d^	4.94 ± 0.07^cd^
	0.25% CR + 2% CH	5.03 ± 0.19^b^	4.52 ± 0.19^c^	4.07 ± 0.23^f^	4.05 ± 0.14^d^	4.79 ± 0.15^d^
	0.5% CR + 2% CH	3.14 ± 0.48^c^	4.37 ± 0.09^cd^	4.38 ± 0.31^ef^	4.13 ± 0.18^d^	4.19 ± 0.33^e^
	1% CR + 2% CH	4.66 ± 0.17^bc^	3.90 ± 0.40^d^	4.25 ± 0.24^ef^	4.33 ± 0.27^cd^	4.54 ± 0.20^de^
2	Baseline	7.29 ± 0.02^a^	6.97 ± 0.10^a^	6.64 ± 0.20^a^	6.71 ± 0.13^a^	6.78 ± 0.13^a^
	BPD control	6.47 ± 0.07^b^	5.64 ± 0.13^b^	5.45 ± 0.07^b^	5.75 ± 0.15^b^	5.87 ± 0.13^b^
	50 mM acetic acid	6.38 ± 0.11^b^	7.07 ± 0.08^a^	5.96 ± 0.17^ab^	5.96 ± 0.07^b^	5.92 ± 0.12^b^
	2% CH	6.08 ± 0.07^bc^	4.81 ± 0.10^d^	4.28 ± 0.09^c^	4.62 ± 0.15^c^	4.46 ± 0.05^cde^
	0.25% CR	5.70 ± 0.22^cd^	5.20 ± 0.02^c^	3.90 ± 0.70^cd^	4.71 ± 0.16^c^	4.93 ± 0.14^c^
	0.5% CR	5.25 ± 0.24^edf^	5.20 ± 0.04^c^	4.23 ± 0.19^c^	4.54 ± 0.12^c^	4.78 ± 0.22^cd^
	1% CR	4.72 ± 0.24^gf^	4.58 ± 0.17^de^	2.96 ± 0.54^d^	4.34 ± 0.21^cd^	4.18 ± 0.13^e^
	0.25% CR + 2% CH	5.31 ± 0.29^ed^	4.45 ± 0.27^de^	4.02 ± 0.34^c^	4.48 ± 0.29^c^	4.22 ± 0.10^e^
	0.5% CR + 2% CH	4.80 ± 0.16^egf^	3.99 ± 0.13^f^	3.86 ± 0.33^cd^	3.93 ± 0.15^d^	4.33 ± 0.28^ed^
	1% CR + 2% CH	4.38 ± 0.32^g^	4.39 ± 0.11^e^	4.44 ± 0.19^c^	3.85 ± 0.24^d^	4.23 ± 0.31^e^


*^*1*^n = 5 replicates per treatment per day per trial. Values (log_*10*_ cfu/sample) presented as mean ± standard error of the mean. Within the same trial, within column with no common superscript differ significantly (*P* < 0.05).*

### Efficacy of GA-Based or CH-Based Coating Treatments (With or Without CR) in Reducing Aerobic Bacterial Counts on Chicken Wingettes

Table 4 shows the effect of GA (10%), CR (0.25, 0.5 or 1%) and their combinations on the total aerobic counts on chicken wingettes. The total aerobic counts recovered from the baseline group was ∼5.34 log_10_ cfu/sample and ∼4.24 log_10_ cfu/sample at day 0 in trial 1 and 2 respectively. By the end of the storage, it was observed that the aerobic counts in baseline group increased by ∼3.33 log_10_ cfu/sample in trial 1 and ∼4.46 log_10_ cfu/sample in trial 2. The BPD washing did not significantly reduce aerobic bacterial load except for minimal reductions of ∼0.5 log_10_ cfu/sample at days 5 and 7 in trial 1 when compared to the baseline. The 10% GA coating did not exert any antimicrobial effect on the aerobic bacteria. The total aerobic counts recovered from the samples coated with 10% GA was similar to baseline, except for minimal reduction at day 7 in trial 1. Among the 3 CR treatments, only 1% CR wash treatment consistently reduced total aerobic counts by ∼0.5 to 1 log_10_ cfu/sample as compared to the BPD control in both trials and all sampling days (except day 5 in trial 1). The combination treatments of GA and CR did not differ in their efficacy in reducing total aerobic counts when compared to either GA or CR on majority of sampling days.

**Table 4:** The efficacy of gum arabic (GA), carvacrol (CR) and their combinations on total aerobic counts on chicken wingettes^1^.

Trial	Treatments	Day 0	Day 1	Day 3	Day 5	Day 7
1	Baseline	5.34 ± 0.18^ab^	5.71 ± 0.31^ab^	7.60 ± 0.31^a^	7.97 ± 0.23^ab^	8.67 ± 0.10^a^
	BPD control	5.22 ± 0.21^ab^	6.30 ± 0.36^a^	7.05 ± 0.24^ab^	7.20 ± 0.13^cd^	8.02 ± 0.22^b^
	10% GA	5.69 ± 0.35^a^	5.87 ± 0.25^ab^	7.22 ± 0.23^ab^	7.48 ± 0.19^bc^	7.98 ± 0.04^b^
	0.25% CR	5.17 ± 0.22^ab^	5.37 ± 0.29^b^	6.98 ± 0.25^ab^	6.55 ± 0.27^e^	7.44 ± 0.03^c^
	0.5% CR	4.66 ± 0.22^bc^	4.37 ± 0.17^c^	6.55 ± 0.32^bc^	6.65 ± 0.11^de^	7.56 ± 0.18^bc^
	1% CR	4.30 ± 0.27^c^	5.00 ± 0.49^bc^	6.04 ± 0.56^cd^	7.33 ± 0.06^c^	7.14 ± 0.05^c^
	0.25% CR + 10% GA	4.76 ± 0.32^bc^	5.12 ± 0.32^bc^	5.91 ± 0.32^cd^	7.51 ± 0.22^bc^	8.53 ± 0.15^a^
	0.5% CR + 10% GA	4.06 ± 0.23^c^	5.65 ± 0.36^ab^	5.60 ± 0.16^d^	7.74 ± 0.29^abc^	7.98 ± 0.04^b^
	1% CR + 10% GA	4.65 ± 0.33^bc^	4.34 ± 0.27^c^	5.91 ± 0.27^cd^	8.12 ± 0.14^a^	7.60 ± 0.19^bc^
2	Baseline	4.24 ± 0.04^bc^	5.32 ± 0.16^cd^	7.38 ± 0.09^ab^	7.09 ± 0.11^a^	8.70 ± 0.07^a^
	BPD control	4.22 ± 0.14^bc^	6.11 ± 0.17^b^	7.74 ± 0.06^a^	6.99 ± 0.07^a^	8.57 ± 0.11^ab^
	10% GA	4.83 ± 0.35^b^	7.14 ± 0.22^a^	7.67 ± 0.13^a^	6.84 ± 0.15^ab^	8.86 ± 0.10^a^
	0.25% CR	3.72 ± 0.28^cd^	5.57 ± 0.22^bcd^	6.90 ± 0.46^b^	6.30 ± 0.11^de^	8.26 ± 0.13^c^
	0.5% CR	3.79 ± 0.12^cd^	5.16 ± 0.17^d^	7.32 ± 0.10^ab^	6.23 ± 0.06^e^	8.37 ± 0.07^bc^
	1% CR	3.50 ± 0.12^d^	5.56 ± 0.18^cd^	6.80 ± 0.15^b^	5.88 ± 0.14^f^	8.17 ± 0.19^c^
	0.25% CR + 10% GA	5.54 ± 0.39^a^	5.73 ± 0.23^bc^	7.65 ± 0.13^a^	6.68 ± 0.08^bc^	8.08 ± 0.09^c^
	0.5% CR + 10% GA	6.18 ± 0.31^a^	5.30 ± 0.14^cd^	7.16 ± 0.21^ab^	6.54 ± 0.09^cd^	8.33 ± 0.10^bc^
	1% CR + 10% GA	5.98 ± 0.17^a^	5.80 ± 0.20^bc^	6.03 ± 0.27^c^	6.33 ± 0.05^de^	8.36 ± 0.07^bc^


*^*1*^n = 5 replicates per treatment per day per trial. Values (log_*10*_ cfu/sample) presented as mean ± standard error of the mean. Within the same trial, within column with no common superscript differ significantly (*P* < 0.05).*

The effect of CH (2%), CR (0.25, 0.5, and 1%) and their combinations on the total aerobic counts on chicken wingettes is presented in Table 5. As observed in Table 4, the total aerobic counts in baseline group increased from day 0 through day 7 by ∼2 to 3 log_10_ cfu in both trials. Unlike the 10% GA, the 2% CH showed significant reduction on the total aerobic counts at all sampling days expect day 3 in trial 1 when compared to either baseline or BPD control. In trial 2, the reduction of aerobic bacteria with 2% CH was consistent throughout all sampling days. When compared to the 50 mM acetic acid solution, the 2% CH significantly reduced the total aerobic counts at days 0, 5, and 7 in trial 1, and days 1 and 3 in trial 2. The combination treatments of CR and CH produced microbial reductions on majority of sampling days (except day 3 in trial 1; days 0 and 7 in trial 2) when compared with the BPD and baseline. However, the combination treatments were not significantly more effective than the respective doses of CR or CH alone for majority of the timepoints.

**Table 5:** The efficacy of chitosan (CH), carvacrol (CR) and their combinations on total aerobic counts on chicken wingettes^1^.

Trial	Treatments	Day 0	Day 1	Day 3	Day 5	Day 7
1	Baseline	7.06 ± 0.12^a^	9.68 ± 0.05^a^	8.16 ± 0.05^a^	9.18 ± 0.02^a^	9.08 ± 0.15^ab^
	BPD control	6.96 ± 0.15^a^	9.56 ± 0.03^ab^	8.18 ± 0.07^a^	9.21 ± 0.13^a^	9.27 ± 0.67^a^
	50 mM acetic acid	6.62 ± 0.18^b^	9.15 ± 0.12^bc^	8.00 ± 0.11^a^	9.09 ± 0.08^a^	9.28 ± 0.16^a^
	2% CH	6.15 ± 0.11^de^	8.74 ± 0.19^cd^	7.55 ± 0.19^ab^	8.32 ± 0.06^cd^	8.59 ± 0.25^cd^
	0.25% CR	6.40 ± 0.07^bcd^	8.55 ± 0.06^d^	8.00 ± 0.07^a^	8.92 ± 0.16^ab^	8.83 ± 0.08^bc^
	0.5% CR	6.01 ± 0.09^e^	8.34 ± 0.03^de^	8.11 ± 0.20^a^	8.85 ± 0.17^ab^	8.20 ± 0.05^e^
	1% CR	6.29 ± 0.14^bcde^	8.64 ± 0.17^d^	7.91 ± 0.49^ab^	8.60 ± 0.10^bc^	8.31 ± 0.06^de^
	0.25% CR + 2% CH	6.58 ± 0.10^bc^	7.93 ± 0.20^ef^	7.50 ± 0.44^ab^	8.56 ± 0.09^bc^	8.32 ± 0.31^de^
	0.5% CR + 2% CH	6.27 ± 0.11^cde^	7.69 ± 0.14^f^	7.02 ± 0.05^b^	7.96 ± 0.21^d^	8.57 ± 0.04^cd^
	1% CR + 2% CH	6.07 ± 0.08^de^	7.04 ± 0.38^g^	7.59 ± 0.33^ab^	8.08 ± 0.19^d^	8.19 ± 0.16^e^
2	Baseline	4.50 ± 0.15^a^	5.73 ± 0.23^a^	6.79 ± 0.24^a^	7.32 ± 0.08^ab^	7.42 ± 0.04^ab^
	BPD control	4.06 ± 0.23^ab^	5.72 ± 0.20^a^	6.02 ± 0.16^ab^	7.45 ± 0.17^a^	7.44 ± 0.10^a^
	50 mM acetic acid	3.78 ± 0.15^bc^	5.16 ± 0.22^b^	5.77 ± 0.18^b^	7.00 ± 0.27^bc^	7.04 ± 0.09^cd^
	2% CH	3.41 ± 0.14^c^	3.84 ± 0.21^de^	4.92 ± 0.26^cd^	6.56 ± 0.16^c^	6.68 ± 0.23^d^
	0.25% CR	4.36 ± 0.14^a^	4.70 ± 0.23^bc^	5.43 ± 0.10^bc^	6.89 ± 0.11^bc^	7.50 ± 0.05^a^
	0.5% CR	3.79 ± 0.17^bc^	4.35 ± 0.23^cd^	5.91 ± 0.13^b^	6.80 ± 0.08^c^	7.45 ± 0.11^a^
	1% CR	3.50 ± 0.18^bc^	3.75 ± 0.16^e^	4.31 ± 0.61^de^	6.79 ± 0.15^c^	7.43 ± 0.13^ab^
	0.25% CR + 2% CH	3.60 ± 0.26^bc^	4.18 ± 0.08^cde^	4.31 ± 0.32^de^	6.79 ± 0.07^c^	7.22 ± 0.12^abc^
	0.5% CR + 2% CH	3.64 ± 0.17^bc^	4.13 ± 0.05^de^	4.24 ± 0.07^de^	6.73 ± 0.18^c^	7.33 ± 0.06^abc^
	1% CR+2% CH	3.31 ± 0.30^c^	4.06 ± 0.14^de^	4.05 ± 0.22^e^	6.58 ± 0.16^c^	7.07 ± 0.20^bc^


*^*1*^n = 5 replicates per treatment per day per trial. Values (log_*10*_ cfu/sample) presented as mean ± standard error of the mean. Within the same trial, within column with no common superscript differ significantly (*P* < 0.05).*

### The Effect of Treatments on the Color of Chicken Wingettes

The treatment of chicken wingettes with GA (10%), 50 mM acetic acid (pH ∼6.5), CH (2%), CR (0.25, 0.5, or 1%), or combinations did not produce significant changes on the color values (L^∗^, a^∗^, b^∗^) of wingettes when compared with either BPD control or baseline group (Table 6) within the same sampling day. The refrigerated storage had no significant effect on the lightness of chicken wingettes (Table 6A). At the end of 7 days of storage, the redness of wingettes did not significantly differ from either day 0 or day 3 in all treatments except baseline and 1% CR (Table 6B). Majority of treatments did not affect yellowness (b^∗^) during refrigerated storage (Table 6C). However, the combination treatments of 0.5, 1% CR with CH and 1% CR with GA decreased the yellowness of wingettes during 7 days of refrigerated storage.

**Table 6:** The effect of gum arabic (GA), chitosan (CH), carvacrol (CR) and their combinations on **(A)** lightness, **(B)** redness, and **(C)** yellowness of chicken wingettes^1^.

(A) Lightness (L^∗^)		

Treatments	Day 0	Day 3	Day 7
Baseline	72.20 ± 0.31	72.76 ± 0.45	71.68 ± 0.38
BPD control	73.16 ± 0.33	73.60 ± 0.22	72.96 ± 0.22
10% GA	71.64 ± 0.59	71.89 ± 0.72	72.17 ± 1.26
50 mM acetic acid	72.68 ± 0.44	72.18 ± 0.68	71.25 ± 0.83
2% CH	72.91 ± 0.43	73.05 ± 0.74	72.48 ± 1.22
0.25% CR	72.61 ± 0.55	73.43 ± 0.57	72.25 ± 0.81
0.5% CR	73.10 ± 0.30	72.65 ± 0.55	72.07 ± 0.61
1% CR	72.93 ± 0.28	73.36 ± 0.32	72.62 ± 0.32
0.25% CR + 10% GA	71.73 ± 1.35	71.89 ± 0.60	71.53 ± 0.44
0.5% CR + 10% GA	72.24 ± 0.52	72.05 ± 0.60	71.46 ± 0.43
1% CR + 10% GA	71.53 ± 0.74	72.03 ± 0.45	71.19 ± 0.59
0.25% CR + 2% CH	73.11 ± 0.92	73.22 ± 1.00	72.70 ± 1.02
0.5% CR + 2% CH	72.50 ± 0.71	73.03 ± 0.71	72.30 ± 0.56
1% CR + 2% CH	73.24 ± 0.88	73.43 ± 0.49	72.55 ± 0.74

*^*1*^n = 5 replicates per treatment per day. Values (mean ± standard error of the mean). No significant different within the same column or within the same row (*P* > 0.05).*			

**(B) Redness (a^∗^)**			

**Treatments**	**Day 0**	**Day 3**	**Day 7**

Baseline	2.98 ± 0.42^b^	3.71 ± 0.14^ab^	3.80 ± 0.23^a^
BPD control	2.98 ± 0.44^a^	2.56 ± 0.27^a^	2.99 ± 0.35^a^
10% GA	4.04 ± 0.42^a^	3.68 ± 0.40^a^	4.03 ± 0.40^a^
50 mM acetic acid	3.81 ± 0.44^a^	3.31 ± 0.29^a^	3.86 ± 0.34^a^
2% CH	3.57 ± 0.26^a^	3.14 ± 0.31^a^	3.63 ± 0.66^a^
0.25% CR	3.48 ± 0.74^a^	2.67 ± 0.37^a^	3.90 ± 0.22^a^
0.5% CR	3.33 ± 0.66^a^	3.35 ± 0.75^a^	4.27 ± 0.28^a^
1% CR	3.79 ± 0.16^a^	3.01 ± 0.14^b^	3.71 ± 0.24^a^
0.25% CR + 10% GA	4.43 ± 0.68^a^	2.93 ± 0.29^b^	3.90 ± 0.60^ab^
0.5% CR + 10% GA	4.38 ± 0.30^a^	3.54 ± 0.35^a^	3.85 ± 0.37^a^
1% CR + 10% GA	4.50 ± 0.82^a^	2.90 ± 0.66^a^	3.76 ± 0.77^a^
0.25% CR + 2% CH	4.44 ± 0.78^a^	3.13 ± 0.64^a^	3.31 ± 0.43^a^
0.5% CR + 2% CH	4.42 ± 0.66^a^	3.24 ± 0.64^a^	3.78 ± 0.51^a^
1% CR + 2% CH	3.79 ± 0.22^a^	3.18 ± 0.29^a^	4.02 ± 0.54^a^

**(C) Yellowness (b^∗^)**			

**Treatments**	**Day 0**	**Day 3**	**Day 7**

Baseline	10.19 ± 0.63^a^	9.30 ± 0.42^a^	9.05 ± 0.83^a^
BPD control	9.62 ± 0.57^a^	8.10 ± 0.24^a^	8.30 ± 0.51^a^
10% GA	9.54 ± 0.33^a^	7.96 ± 0.50^a^	8.80 ± 0.60^a^
50 mM acetic acid	9.65 ± 0.49^a^	8.61 ± 0.33^a^	8.33 ± 0.74^a^
2% CH	10.32 ± 0.73^a^	8.66 ± 0.96^a^	8.82 ± 0.94^a^
0.25% CR	10.01 ± 1.70^a^	8.10 ± 1.50^a^	9.18 ± 1.04^a^
0.5% CR	10.40 ± 1.25^a^	9.60 ± 0.96^a^	9.51 ± 1.09^a^
1% CR	11.27 ± 0.82^a^	9.30 ± 1.00^a^	9.08 ± 1.11^a^
0.25% CR + 10% GA	11.97 ± 1.36^a^	9.73 ± 0.59^a^	9.59 ± 1.37^a^
0.5% CR + 10% GA	10.78 ± 0.79^a^	8.03 ± 0.94^a^	8.41 ± 1.27^a^
1% CR + 10% GA	10.98 ± 0.37^a^	8.66 ± 1.09^b^	8.97 ± 0.68^b^
0.25% CR + 2% CH	10.72 ± 0.69^a^	8.60 ± 1.00^a^	9.23 ± 0.71^a^
0.5% CR + 2% CH	11.82 ± 0.74^a^	9.62 ± 0.56^b^	9.98 ± 0.32^b^
1% CR + 2% CH	11.55 ± 0.66^a^	8.56 ± 0.71^b^	9.06 ± 0.55^b^


*^*1*^n = 5 replicates per treatment per day. Values (mean ± standard error of the mean). No significant different within the same column (*P* > 0.05). Means with no common letter differ significantly (*P* < 0.05) within the same row.*

### Gene Expression Profile of *C. jejuni* in Response to Sub-Inhibitory Concentration of CR, CH, and Their Combination

**FIGURE 1:**
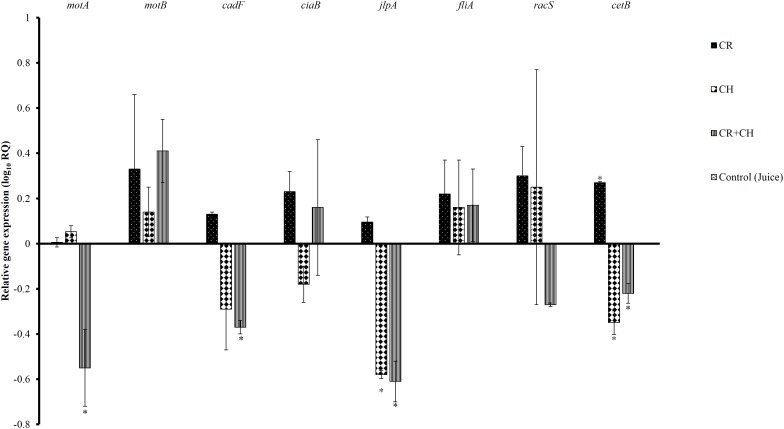
The effect of 0.002% carvacrol (CR), 0.0125% chitosan (CH) and 0.002% CR + 0.0125% CH on the expression of selected virulence genes of *C. jejuni S-8.* 16S-rRNA served as endogenous control. Results are averages of two independent experiments, each containing duplicate samples (mean and SEM). ^∗^Indicates significantly down or up-regulated genes (*P* < 0.05).

The sub-inhibitory concentration of CR and CH was determined using growth curves (data not shown). Figure 1 shows the effect of sub-inhibitory concentration of CR, CH, and their combination on the expression of selected *C. jejuni* genes required for survival and virulence in the host. The presence of CR at the sub-inhibitory concentration significantly up-regulated energy taxis gene, *cetB*. However, CH and CH + CR combination significantly down-regulated the expression of *cetB*. The expression of motility gene *motA* and fibronectin binding protein gene *cadF* was significantly downregulated by CH + CR combination treatment but not by CR and CH treatments alone. The gene *jlpA* that helps in bacterial attachment was also reduced in expression by CH + CR combination and CH treatment. The expression of motility gene (*motB*), invasion antigen protein gene (*ciaB*), flagella biosynthesis RNA polymerase sigma protein gene (*fliA*) and regulatory protein gene (*racS)* were not affected by any of the treatment groups when compared to the control. The acetic acid treatment did not affect the expression of tested genes (*P* > 0.05).

## Discussion

In this study, we evaluated the potential of GA or CH-based edible coatings fortified with CR to reduce *C. jejuni* on chicken wingettes. The wingettes were used as a model to represent the treatment of a whole carcass. The increased contact time between coating and the product facilitates in reducing pathogen survival and also prevents subsequent contamination during transport and post-coating handling. Both GA and CH have been extensively studied as coatings on food products such as fresh strawberries, longan fruit, tomato, skinless frankfurters, and shell eggs to improve food safety (El Ghaouth et al., 1991; Jiang and Li, 2001; Ali et al., 2010; Upadhyay et al., 2015; Upadhyaya et al., 2016). GA dissolves in water at neutral pH while CH needs an acidified solution. Therefore, aqueous solution of acetic acid at 50 mM concentration was used to dissolve the CH powder. The gum arabic coating significantly reduced *C. jejuni* counts when compared to non-coated wingettes (baseline). However, GA efficacy was similar to BPD wash treatment suggesting that reductions observed in GA treatment were probably due to removal of loosely attached *C. jejuni* cells (Table 2). Similar results were previously reported where 10% GA coating did not inhibit mycelia growth of *Colletotrichum musae* and *Colletotrichum gloeosporioides* in artificially inoculated bananas and papayas (Maqbool et al., 2011). Likewise, Jiang et al. (2013) observed that *Pseudomonas* spp., yeasts and molds counts were not significantly reduced by the 10% GA coating on mushrooms. Upadhyaya et al. (2016) also observed that the presence of GA coating did not exert any antimicrobial effect on *Salmonella* present on eggs as compared to controls. In contrast to GA, coatings with the 2% CH showed significant antimicrobial activity against *C. jejuni* on wingettes (Table 3). Olaimat et al. (2014) observed that 2% CH and 0.2% κ-carrageenan combination coating containing mustard extract significantly reduced (up to 2.78 log_10_ cfu/g) *C. jejuni* counts on chicken breast. In the present study, coating materials fortified with select concentrations of CR significantly improved the antimicrobial activity of coating agents. Moreover, the antimicrobial efficacy was maintained through 7 days of refrigerated storage. Similar results have been observed in previous studies where the addition of CR to GA (Upadhyaya et al., 2016) or to CH (Upadhyay et al., 2015) significantly improved the antimicrobial efficacy of coating material against *S.* Enteritidis on shell eggs and *L. monocytogenes* on frankfurters, respectively. The presence of CR wash treatments also reduced *C. jejuni* counts on wingettes and the efficacy of several CR wash treatments was similar to the coating treatments with GA or CH. However, since wash treatments do not exert any antimicrobial effect after initial treatment, protection from subsequent contamination event during handling or storage is a potential concern.

The presence of aerobic bacteria such as *Pseudomonas* spp., in refrigerated chicken products can reduce shelf–life and decrease food safety (De Ledesma et al., 1996; Huis in’t Veld, 1996; Kim and Marshall, 2000). In this study, we observed that direct application of CR or CH + CR coatings inhibited the growth of total aerobic counts when compared with either non-coated or wingettes washed with BPD control. Previously, Siripatrawan and Noipha (2012) observed that 2% CH reduced the count of total aerobic bacteria, yeast, molds and lactic acid bacteria from day 12 through 20 on pork sausages stored at 4°C. Aşik and Candoğan (2014) found that 3% CH alone or in combination with different concentrations (0.5, 1, and 1.5%) of garlic oil significantly reduced the total aerobic counts on shrimp and improved the shelf-life of products by at least 2 days. In agreement with these studies, a recent study conducted by Jasour et al. (2015) reported that CH-based coatings supplemented with lactoperoxidase extended the shelf-life of trout filets by at least 4 days. Chicken skin harbors different microflora, including psychotropic bacteria that contributes to spoilage (Mead, 2004). In the present study, an increase in the total aerobic counts was observed throughout the sampling days in all treatments, which could be due to the growth of those psychrophiles attached to the skin surfaces of wingettes.

Since color of the poultry product is one of the main attributes of the product which may influence the purchasing decision for consumers, we evaluated the effect of CR and combination treatments on the color of chicken wingettes. Among L^∗^, a^∗^ and b^∗^ values, L^∗^ is the most important since consumers can easily detect the changes in lightness of the products which could affect their purchasing decisions (Guidi and Castigliego, 2010). We observed that there were no significant differences in color values (L^∗^, a^∗^, b^∗^) of chicken wingettes between treatments and controls within the sampling day (Tables 6A–C). Previous studies have shown that pH of the product is one of the factors that determines the color of the product (Livingston and Brown, 1981; Yang and Chen, 1993; Allen et al., 1997). In this study, the pH of treatment solutions, including controls was ∼6.5 to ∼6.7. This could be one of the reasons why a change in color of wingettes within the same sampling day was not observed. Moreover, we observed that refrigerated storage did not affect the lightness or redness of samples. However, a slight decrease in the yellowness of the samples treated with the coating combinations of either GA with 1% CR or CH with 0.5 and 1% CR was observed.

The sub-inhibitory concentration of a compound refers to the maximum concentration that does not inhibit pathogen growth. Our previous studies (Arambel et al., 2015; Upadhyay et al., 2017; Wagle et al., 2017a,b) and other researchers (Castillo et al., 2014; Oh and Jeon, 2015; Kovács et al., 2016) have reported that sub-inhibitory concentrations of natural compounds including phytochemicals modulate the expression of virulent genes in major pathogens including *C. jejuni* thereby potentially changing their pathophysiology and survival efficacy in the environment. To delineate the potential mechanism of action of the tested natural compounds against *C. jejuni*, we evaluated the effect of sub-inhibitory concentrations of CR (0.002%), CH (0.0125%), and CH + CR combination on the expression of *C. jejuni* genes critical for survival and virulence. Since 10% GA did not reduce *C. jejuni* counts on chicken wingettes when compared to the BPD control, we did not include GA as a treatment in gene expression study. To represent the chicken meat environment, gene expression analysis was carried out in chicken meat juice (5% vol/vol) since chicken meat juice is known to enhance surface attachment of *C. jejuni* (Brown et al., 2014), thereby, enhancing their survival in the poultry products (Birk et al., 2004).

Several recent studies have identified genes responsible for *C. jejuni* virulence, colonization in the chicken gut, and infection in humans (Dasti et al., 2010; Hermans et al., 2011). *C. jejuni* genes *motA*, *motB, fliA* are essential for motility (Ketley, 1997; Young et al., 2007). The *cetA* and *cetB* genes are responsible for energy taxis (Hendrixson et al., 2001). In addition, *cadF* and *jlpA* are required for cell surface attachment (Jin et al., 2003; Hermans et al., 2011). In this study, we observed that the CR treatment did not significant change the expression of a majority of test genes except *cetB.* In contrast, CH down-regulated the expression of *cetB* and *jlpA* suggesting that the two compounds could be acting by different mechanisms. The CH + CR combination significantly downregulated the expression of *motA*, *cadF, jlpA* and *cetB*, indicating that the combination treatments could modulate *C. jejuni* virulence and capacity to cause infections in humans.

## Conclusion

In the present study, GA or CH-based coating with CR produced consistent reduction of *C. jejuni* on chicken wingettes. The aforementioned reduction would have significant food safety implications since a 2-log_10_ reduction of *Campylobacter* from the poultry carcass could result in a 30-fold reduction in human infection (Rosenquist et al., 2003). In addition, the aforementioned treatments also reduced the expression of selected virulence genes of *C. jejuni*. Thus, CR in combination with GA, CH coating could be used as an effective antimicrobial treatment for controlling *C. jejuni* and improving safety of poultry products, however, follow up investigations testing the effect of treatments on the organoleptic properties of products is warranted.

## Data Availability

All datasets generated for this study are included in the manuscript.

## Author Contributions

SS and DD designed the study. SS, BW, KA, IU, and AU conducted the experiments. SS wrote the manuscript. AU, DD, and AD critically analyzed and revised the manuscript.

## Disclaimer

Mention of a trade name, proprietary product, or specific equipment does not constitute a guarantee or warranty by the USDA and does not imply its approval to the exclusion of other products that may be suitable.

## Conflict of Interest Statement

The authors declare that the research was conducted in the absence of any commercial or financial relationships that could be construed as a potential conflict of interest.
